# Molecular Cloning and Characterization of a *Fasciola gigantica* Nuclear Receptor Subfamily 1 (FgNR1)

**DOI:** 10.3390/pathogens11121458

**Published:** 2022-12-01

**Authors:** Pongsakorn Martviset, Pathanin Chantree, Salisa Chaimon, Nattaya Torungkitmangmi, Parisa Prathaphan, Jittiporn Ruangtong, Phornphan Sornchuer, Nattaya Thongsepee, Kant Sangpairoj, Poom Adisakwattana

**Affiliations:** 1 Department of Preclinical Science, Faculty of Medicine, Thammasat University, Pathumthani 12120, Thailand; 2 Thammasat University Research Unit in Nutraceuticals and Food Safety, Thammasat University, Pathumthani 12120, Thailand; 3 Research Group in Medical Biomolecules, Faculty of Medicine, Thammasat University, Pathumthani 12120, Thailand; 4 Graduate Program in Biochemistry and Molecular Biology, Faculty of Medicine, Thammasat University, Pathumthani 12120, Thailand; 5 Department of Helminthology, Faculty of Tropical Medicine, Mahidol University, Bangkok 10400, Thailand

**Keywords:** *Fasciola gigantica*, FgNR1, nuclear receptor, drug target

## Abstract

*Fasciola gigantica*, a giant liver fluke, causes tremendous loss to the livestock economy in several regions throughout the world. The situation of drug resistance has been emerging increasingly; therefore, novel drugs and drug targets need to be discovered. The adult *F. gigantica* inhabits the major bile ducts where bile salts accumulate—these are steroid-like molecules that mediate several physiological processes in organisms through interacting with their specific nuclear receptors. However, the molecular mechanism of the interaction in the parasitic organisms have not been clearly understood. In this study, putative nuclear receptor subfamily 1 of *F. gigantica* (FgNR1) was identified. Nucleotide and amino acid sequences of the FgNR1 homolog were obtained from the transcriptome of *F. gigantica* and predicted for properties and functions using bioinformatics. The full-length cDNA was cloned and expressed in the bacterial expression system and then used for immunization. Western analysis and immunolocalization suggested that FgNR1 could be detected in the crude worm antigens and was highly expressed in the caeca and testes of the adult parasite. Moreover, the bile could significantly activate the expression of FgNR1 in cultured parasites. Our results indicated that FgNR1 has high potential for the development of a novel anthelminthic drug in the future.

## 1. Introduction

*Fasciola* spp., the liver fluke, is commonly found in ruminants such as cows, water buffaloes, goats, and sheep [1]. The infection causes severe complications that are reflected in a reduction in the productivity of the dairy and meat industries, which has a high impact on economic losses. Not only livestock, but humans are also susceptible and present clinical manifestations either similar to animals, or even more severe because of extrahepatic migration [2]. The distribution of *Fasciola* spp. covers approximately 75 countries, with more than seven hundred million animals and 2.4 million people predicted to be infected [3]. There are two major species: the first is *Fasciola hepatica*, which are widely spread throughout the globe; especially in countries in South America and the Middle East. Secondly, infection with *F. gigantica* in ruminants is the major veterinary problem in the tropical areas of Africa, Southern Asia, and Southeast Asia including Thailand [3]. The parasite generally transmits to the host via the ingestion of an infective metacercaria attached to water plants, followed by passing through the small intestine where it excysts. The newly excysted juvenile (NEJ) penetrates the small intestinal wall, passes through the peritoneal cavity, and then inhabits the liver parenchyma as well as the large bile ducts, where they develop to the adult stage and lay eggs. Inside the bile duct, the parasite is surrounded by the bile components that provide nutrients, bile salts, inorganic salts, cholesterols, hormones, etc. [1,4]. 

Triclabendazole remains a standard anthelminthic drug for the treatment of animal and human fasciolosis, but drug resistance has been increasingly reported in several countries throughout the globe [5,6,7,8]. Even though many researchers in several countries have tried to combine triclabendazole with other drugs, such as albendazole, to kill the parasite, they currently remain unsuccessful; moreover, the formulated combination has also been resisted [9,10]. 

Bile salts are steroid acids produced and secreted from the liver of mammals and also vertebrates and then conjugated with taurine or glycine mostly for the function of digesting dietary fats and oils [11]. Furthermore, bile salts are the key molecules acting as steroid hormones that interact with their specific receptors, especially nuclear receptors, and then trigger transcriptional processes inside the nucleus and facilitate intracellular signaling cascades [12].

Nuclear receptors (NRs) are a group of proteins that accumulate in the nucleus of the eukaryotic cells that act as transcription factors regulating many cell activities [13]. Previous studies demonstrated the major functions of NRs in many aspects, such as eukaryotic cell development, differentiation, reproduction, and metabolic homeostasis [13,14]. NRs are classified into six families. Family 1, the largest family, or steroid receptor family, comprises many receptors that respond to steroid hormones, such as thyroid hormone receptor, retinoic acid receptor (RAR), peroxisome proliferator-activated receptor (PPAR), vitamin D receptor, and farnesoid X receptor. This family is also subclassified into subfamily 1A to 1J by using structural conservation and kinds of activating ligands [12,13,15]. Family 2 is a retinoid family that contains principal members, such as hepatocyte nuclear factor-4, retinoic X receptor (RXR), and testicular receptor. Most family 2 members need to interact with retinoid X receptor (RXR) to form a heterodimer and be able to function [12]. The third family belongs to the estrogen receptor family which comprises, for example, estrogen receptor, estrogen-related receptor, androgen receptor, glucocorticoid receptor, mineralocorticoid receptor, progesterone receptor, and some unclear orphan receptors. Family 4 to 6 are the small groups of nuclear receptors that have newly identified but unclear functions [13,15]. Generally, NRs structure contains six domains. The activation function domain-1 (AF-1) is located at almost every N-terminus followed by the DNA binding domain (DBD). DBD is highly conserved among NRs that consists of zinc finger proteins forming two helices, P-box and D-box. DBD recognizes the hexanucleotide response elements within the nuclear receptor-regulated promotors and also acts as an allosteric transmitter to the other domains in the NR [16]. DBD is connected to the hinge domain that is located upstream to the ligand binding domain (LBD). LBD is another conserved domain among NRs, especially in the same subfamily. LBD contains an internal binding pocket that specifically binds to its cognate ligands. Next to LBD, the second AF domain (AF-2) is located here, which is called a ligand-regulated transcriptional activation function as it is necessary for recruiting various coactivating elements [16,17]. The last domain is a C-terminus variable domain that has an unclear function. Several ligands have been reported for NRs activation especially steroid hormones, including bile salts [12,13,15,18].

In the parasites, there is some evidence demonstrating a necessary view of bile in parasite survival, such as supplement growth and enhanced membrane lipid uptake in *Giardia duodenalis* [19,20,21]. Bile also affects oviposition and cell proliferation in *Schistosoma mansoni* with unknown mechanisms [22,23]. Apart from the bile interactions, several NRs have been reported in parasitic organisms. Two thyroid receptors were firstly identified from *S. mansoni* and later three predicted novel NRs containing two DBDs were isolated [24,25]. Moreover, NRs from *Schistosoma japonicum*, *Schistosoma haematobium*, and *Echinococcus* spp. were identified [26,27,28]. The well-characterized studies were established in *Caenorhabditis elegans*, a roundworm model organism, and illustrated an effect of bile salts on modulation of the parasite life span through a specific nuclear receptor– DAF-12 [29,30,31]. DAF-12 was also characterized in *Strongyloides stercoralis* together with an endogenous ligand which probably developed to be an anthelminthic agent [32,33]. For those reasons, NRs will be emerging drug targets for parasitic diseases [28,34,35].

In *Fasciola* spp., the genetic identification, molecular properties, and functions of NRs as well as the interaction between bile salts and their receptors have not been mentioned elsewhere. Therefore, this study would like to address the fundamental properties of *F. gigantica* nuclear receptors subfamily 1 (FgNR1). These findings may lead to an in-depth understanding of the basic biology of *F. gigantica*, especially inside the liver biliary. Additionally, FgNR1 will be a probable novel drug target for developing an effective anthelminthic drug combating drug-resistant *Fasciola* in the future.

## 2. Materials and Methods

### 2.1. Parasites 

Adult *F. gigantica* were collected from naturally infected cattle sacrificed at the local slaughterhouses located in Pathumthani province, central Thailand. The parasites were handled as previously described [36]. In brief, the freshly collected worms were cleaned several times with 0.85% NaCl to remove bile components and tissue debris. For RNA isolation and crude worm antigens extraction, the worms were immediately kept in liquid nitrogen until use. For the preparation of excretory/secretory (ES) products, fresh worms were used. For paraffin embedding, the fresh worms were cut into small pieces and fixed in 4% paraformaldehyde in PBS, pH 7.4. 

### 2.2. Bioinformatic Analysis

The full-length cDNA encoding FgNR1 was obtained from the transcriptome of *F. gigantica* (Genbank accession number: TPP57711.1). The deduced amino acid sequence of FgNR1 was submitted to BLASTP for determining the homologue percentage and identity compared to other orthologs. In addition, the amino acid sequence was subjected to predict basic properties including molecular weight and isoelectric point (EMBOSS Pepstats) [https://www.ebi.ac.uk/Tools/seqstats/emboss_pepstats/] (accessed on 29 September 2022) [37], signal peptide (SignalP 5.0) [38], transmembrane domain (TMHMM server v. 2.0) [39], N- and O-glycosylation sites (NetNGlyc 1.0 and NetOGlyc 4.0) [40], and disulfide bonds (DIpro) [41]. The conserved motifs and consensus residues of FgNR1 were compared with orthologs using Clustal Omega [42]. The evolutionary relationship between FgNR1 and orthologs was analyzed by using a phylogenetic tree and comparative heatmap. The phylogenetic tree was generated by MEGA11 [43] using the maximum likelihood method with 1000 bootstrap replication. The comparative heatmap was constructed using Ident and Sim program [44]. All sequences used in this study are detailed in Appendix A Table A1.

The two-dimensional structure of FgNR1 was predicted using the PDBsum program [45] and the structural image was created using the Polyview method [46]. A three-dimensional structure simulation of FgNR1 was performed by I-TASSER and GalaxyRefine server [47,48] using the template crystal structure of human Retinoic acid receptor RXR-alpha (PDB ID: 4NQA); the structural image was generated using iCn3D [49,50].

### 2.3. Total RNA Isolation and Synthesis of FgNR1 Complementary DNA

Total RNA was extracted from adult *F. gigantica* by using TRIzol reagent (Invitrogen, Carlsbad, CA, USA) using tissue homogenizer following the manufacturer’s instructions. The concentration of total RNA was measured by using a Nanodrop analyzer (ND-2000, Thermo Fisher Scientific, Wilmington, Germany). The isolated total RNA was subsequently treated with RNase-free DNaseI (1 U of DNaseI/ μg of total RNA; Thermo Fisher Scientific, Vilnius, Lithuania) at 37 °C, for 10 min to remove contaminating genomic DNA. The cDNA of FgNR1 was reverse transcribed by RevertAid First Strand cDNA Synthesis Kit (Thermo Fisher Scientific, Vilnius, Lithuania). The full-length FgNR1 cDNA was then amplified from the first strand cDNA template using a specific primer as follows: forward primer (Fw) 5′- GGA TCC ATG AAA CCA AGC CTT ATT CTA-3′ and reverse primer (Rv) 5′-AAG CTT TTC AGT ATG GAC GAA GTA TAA-3′ which incorporated with *Bam*HI and *Hind*III recognition sites (underlined), respectively. PCR amplifications were carried out by using GoTaq^®^ Colorless Master Mix (Promega Corporation, Madison, WI, USA) in a thermal cycler (Mastercycler nexus Eppendorf flexlid, Eppendorf, Germany). The amplification steps included initial denaturation at 95 °C for 5 min, followed by 35 cycles of denaturation at 95 °C for 1 min, annealing at 55 °C for 1 min, extension at 72 °C for 2 min, and one cycle of a final extension at 72 °C for 10 min. The PCR products were size separated on 1% agarose gel containing ViSafe Red Gel Stain (Vivantis, Shah Alam, Malaysia) using 1X TBE buffer at 100 V for 1 h. The PCR product was eluted from agarose gel (PureLink™ Quick Gel Extraction Kit, Invitrogen, Carlsbad, CA, USA) and ligated into pGEM-T Easy vector (Promega Corporation, Madison, WI, USA) and transformed into XL1-Blue *E. coli* competent cells. The cDNA sequence was confirmed for their conformation by a DNA sequencing service (Solgent Co., Ltd., Daejeon, Republic of Korea).

### 2.4. Molecular Cloning of FgNR1 

The full-length FgNR1 cDNA fragment was digested from pGEM-T Easy by using *Bam*HI/*Hind*III restriction enzymes (Fastdigest restriction enzymes, Thermo Fisher Scientific, Lithuania), and the digested products were eluted (PureLink™ Quick Gel Extraction Kit, Invitrogen, Carlsbad, CA, USA), and subcloned into pET32a(+) (Novagen, EMD Chemicals Inc., Darmstadt, Germany). The recombinant plasmid was then chemically transformed using the heat shock method into BL21(DE3) *E. coli* competent cells. The positive transformants were selected by direct colony PCR using a specific primer as previously mentioned. 

### 2.5. Expression and Purification of Recombinant FgNR1 (rFgNR1) and Recombinant Thioredoxin (rTrx) Fusion Proteins

Recombinant protein rFgNR1 was expressed simultaneously with Trx fusion protein from the pET32a(+) vector. rFgNR1+Trx expression was induced by adding isopropyl β-d-1-thiogalactopyranoside (IPTG, Sigma-Aldrich, Saint Louis, MO, USA) to a final concentration of 1 mM at 37 °C for 1 to 4 h and the solubility of rFgNR1+Trx was also evaluated. The expression of 3 h after induction was selected for the further purification process. For rFgNR1+Trx purification, bacterial cells were harvested by centrifugation at 6000× *g* at 4 °C for 30 min and the pellet was resuspended in lysis buffer (8 M urea, 100 mM NaH_2_PO_4_, 10 mM Tris-Cl, pH 8.0) and allowed to complete lysis for 1 h at 4 °C with agitation. The homogenates were centrifuged at 14,000× *g* at 4 °C for 30 min. The supernatant was collected and mixed with Ni-Sepharose^®^ high performance (Cytiva, Uppsala, Sweden) and rotated at 4 °C for 1 h. The resins were loaded into a polypropylene column and purified by gravity flow under denaturing condition according to the manufacturer recommendations. The purified rFgNR1+Trx was size separated on 12.5% SDS-PAGE and verified by Western analysis with mouse anti-Histidine tag antibody (Bio-Rad Laboratories Inc., Hercules, CA, USA). The recombinant thioredoxin (rTrx) was synthesized from the same expression system by using BL21(DE3) containing pET32a(+) and purified by Ni Sepharose^®^ high performance (Cytiva, Uppsala, Sweden). The purified rFgNR1+Trx and rTdx were dialyzed against PBS, pH 7.4 and concentrated by using 3 kDa Vivaspin™ ultrafiltration spin columns (Cytiva, Buckinghamshire, UK), the concentration was measured by BCA protein assay (Thermo Fisher Scientific, Rockford, IL, USA), and used for raising polyclonal antibodies in mice.

### 2.6. Production of Polyclonal Antibodies against rFgNR1 and rTdx

ICR mice at 6 to 8 weeks of age were immunized three times at 2 week intervals by intraperitoneal injection. A week before the first injection, pre-immunization sera were collected. A total of 100 μg of each purified rFgNR1+Trx or rTrx were mixed thoroughly with TiterMax^®^ Gold adjuvant (Sigma-Aldrich, St. Louis, MO, USA) and injected into mice as the primary injection. After 2 and 4 weeks, the sera were collected and mice were injected with 50 μg of recombinant proteins as the second and third injections, respectively. The final sera were collected 2 weeks after the final injection by cardiac puncture. The specific antibody titer against rFgNR1-Trx and rTrx of each mouse was determined by indirect ELISA using goat anti-mouse IgG antibody, HRP-conjugated (Sigma-Aldrich, St. Louis, MO, USA), and detecting with 1-Step™ Ultra TMB-ELISA Substrate Solution (Sigma-Aldrich, St. Louis, MO, USA).

### 2.7. Preparation of Parasite Protein Extracts

Crude worm antigens (CWA) were prepared from adult parasites by homogenization in the extraction buffer (PBS pH 7.4, 150 mM NaCl, 0.5% Triton X-100, 1 mM PMSF, and 1 mM EDTA) as previously described [51]. The homogenate was centrifuged at 12,000× *g* at 4 °C for 30 min and the supernatant was collected as soluble CWA (s-CWA). The pellet was then resuspended in solubilizing buffer (50 mM Tris-Cl, pH 8.0 and 3% SDS), incubated at 37 °C for 1 h, and centrifuged at 12,000× *g* at 4 °C for 30 min for collecting the supernatant as insoluble CWA (i-CWA). The excretory-secretory (ES) product was prepared from live parasites. After cleaning, adult *F. gigantica* were cultured in PBS, pH 7.4 at 37 °C under 5% CO_2_ atmosphere for 4 h. Parasite eggs and insoluble debris were removed by centrifugation at 4000× *g* for 30 min. The ES products were concentrated by using 3 kDa Vivaspin™ ultrafiltration spin columns (Cytiva, Buckinghamshire, UK). The concentration of protein extracts was determined by Bradford protein assay (PanReac Applichem, ITW reagents, Darmstadt, Germany).

### 2.8. Western Analysis

Mouse polyclonal antibodies against rFgNR1+Trx and rTrx were used for the determination of the native protein, FgNR1, in the parasite extracts. An amount of 100 ng of each rFgNR1+Trx or rTrx, 30 μg of each s-CWA and i-CWA, and 10 μg ES of *F. gigantica* were size separated on 12.5% SDS-PAGE. The proteins were then transferred onto the nitrocellulose membrane (Amersham Protran, Cytiva, Piscataway, NJ, USA) by semidry transferring system (Invitrogen™ Power Blotter, Thermo Fisher Scientific, Carlsbad, CA, USA). Non-specific bindings were blocked using 5% skim milk (Sigma Aldrich, Darmstadt, Germany) for 1 h at room temperature, mouse serum was added in the dilution of 1:400 in antibody diluent (1% BSA in TBS) and incubated at 37°C for 1 h. The membrane was washed several times and then the goat anti-mouse IgG (H+L) secondary antibody, AP conjugate (Invitrogen, Carlsbad, CA, USA), was added and incubated at room temperature for 1 h. Finally, BCIP/NBT substrate was added to the membrane and incubated for a while until the signal could be observed.

### 2.9. Immunolocalization of FgNR1

The tissue section and immunolocalization were performed according to the previous study with some modifications [51]. In summary, fresh collected adult *F. gigantica* were cut into small pieces, fixed in 4% paraformaldehyde, and dehydrated with a serial percentage of ethyl alcohol prior to embedding into paraffin. The fixed tissues were sectioned at 8 μm thickness using microtome (Leica RM 2235, Nussloch, Wetzlar, Germany). For determination of FgNR1 in the parasite tissues, mouse polyclonal antibodies against rFgNR1+Trx or rTrx were used. The sections were dewaxed in xylene twice for 10 min each then rehydrated by ethyl alcohol series ranging from 100 to 70% for 10 min each. The slides were rinsed with tap water and proceeded to the epitope retrieval step. The epitopes were retrieved by immersing in epitope retrieval solution (10 mM Na_3_C_6_H_5_O_7_, pH 6.0 with 0.05% Tween^®^ 20) and heating in microwave oven for 5 min. The buffer was allowed to cool at room temperature for 20 min and then was rinsed with washing buffer (PBS, pH 7.4 with 0.1% Tween^®^ 20) and followed by incubating in glycine blocking solution (0.1% glycine in PBS, pH 7.4) for 30 min. Non-specific bindings were blocked using 4% BSA for 1 h at room temperature, then mouse serum was added to the sections at the dilution of 1:200 in antibody diluent (1% BSA in PBS) and incubated at 4 °C for overnight in a humid chamber. The sections were washed several times and internal peroxidase was blocked by incubating with 3% hydrogen peroxide for 30 min, and then was rinsed with tap water. Rabbit anti-mouse IgG (H+L) secondary antibody, Biotin (Thermo Fisher Scientific, St. Louis, MO, USA) was added and incubated at room temperature for 30 min. ABC peroxidase (Thermo Fisher Scientific, Rockford, IL, USA) was then added and incubated at room temperature for 30 min. Finally, the AEC substrate (Thermo Fisher Scientific, Rockford, IL, USA) was added to the sections and incubated in the dark until the signals were developed.

### 2.10. Parasite Culture with Bile 

The freshly collected worms were cleaned as previously described [36], then washed twice with PBS, pH 7.4, and transferred to recover in a pre-warmed RPMI-1640 culture medium (Gibco, Thermo Fisher Scientific, Carlsbad, CA, USA) without serum and antibiotics at 37 °C under 5% CO_2_, for 30 min. Five intact worms were immediately collected after recovery, washed twice with pre-warmed PBS and total RNA was extracted from each worm using TRIzol reagent (Invitrogen, Carlsbad, CA, USA) according to the manufacturer’s instructions. Other sets of five worms were cultured as previously described [36] in RPMI-1640 medium supplemented with 10% FBS with 0.5 μg/mL and 2 μg/mL of bovine bile (Sigma Aldrich, Darmstadt, Germany) at 37°C under a 5% CO_2_ atmosphere for 4 and 8 h. Finally, worms were collected and proceeded to total RNA extraction.

### 2.11. Real-Time RT-PCR

One µg from each RNA sample was treated with RNase-free DNaseI (Thermo Fisher Scientific, Vilnius, Lithuania) at 37 °C for 10 min and then converted to cDNA using cDNA synthesis kit (Thermo Fisher Scientific, Vilnius, Lithuania) with oligo(dT)18 and random hexamer primer (Sigma-Aldrich, St Louis, MO, USA) according to manufacturer instructions. The cDNA synthesis was terminated by heating at 70 °C for 10 min followed by adding stop reagent, and concentration was measured using a NanoDrop™ 2000 Spectrophotometers (Thermo Fisher Scientific, Wilmington, DE, USA). An amount of 200 ng of cDNA was used as template for qPCR using iTaq™ Universal SYBR^®^ Green Supermix (Bio-Rad Laboratories Inc., Hercules, CA, USA) in a StepOne™ Real-Time PCR Systems (Applied Biosystems, Foster City, CA, USA). Primer for FgNR1 qPCR is as follows: (Fw) 5′-AAT AGA CTC ACG CGT TCT-3′ and (Rv) 5′-AAA GGT TTC GCC GAT ACT AT-3′. *F. gigantica* tubulin (FgTUB) was used as the internal control for calculating fold changes in expression and was amplified in parallel to each set of experiments. The primer for FgTUB is as follows: (Fw) 5′-TGA AGC CTG G GC TCG TTT GGA CCA CAA-3′, (Rv) 5′-TTA GTA TTC TTC ACC CTC GCC TTC ACC-3′. The heat shock protein 70 (HSP70, GenBank: EF506931) was used as the irrelevant target. The primer for FgHSP70 is as follows: (Fw) 5′- GGA TGT GGC ACC TCT TTC AT-3′, (Rv) 5′-AGC TCA AAC TTT CCG AGC AA-3′. qPCR conditions were composed of denaturing at 95 °C for 1 min and then amplified for 40 cycles at the following conditions: 95 °C for 30 s, 60 °C for 30 s and 72 °C for 1 min, followed by a final heating at 72 °C for 3 min. Amplifications were performed in triplicate. Quantification of the gene expression levels was performed using the 2^−∆∆CT^ method [36,52].

## 3. Results

### 3.1. Molecular Properties of FgNR1 

Sequence bioinformatics of FgNR1 demonstrated that its amino acid coding sequence comprises 523 amino acid residues which are translated from 1569 nucleotides. It is predicted to have a molecular weight of about 58.29 kDa with isoelectric point (PI) of 8.0250. The signal peptide prediction by SignalP 5.0 revealed that FgNR1 is absent of signal peptide as it is manipulated in the nucleus. Moreover, FgNR1 does not have a transmembrane domain. The Prediction of disulfide bond formation suggested that FgNR1 has 18 cysteine residues which formed 7 disulfide bonds at C_181_-C_219_, C_308_-C_349_, C_10_-C_27_, C_406_-C_427_, C_46_-C_62_, C_52_-C_65_, C_248_-C_259_. A total of 4 potential N-glycosylation sites were predicted at N_190_, N_232_, N_266_, and N_323_, while 29 potential O-glycosylation sites were predicted at S_7_, S_45_, T_99_, S_101_, N_106_, T_109_, T_114_, S_136_, S_164_, D_180_, L_187_, Q_189_, D_192_, G_201_, S_202_, P_226_, P_230_, S_233_, S_234_, T_238_, A_241_, P_242_, A_244_, S_252_, S_253_, W_268_, T_269_, H_272_, and G_300_.

**Figure 1:**
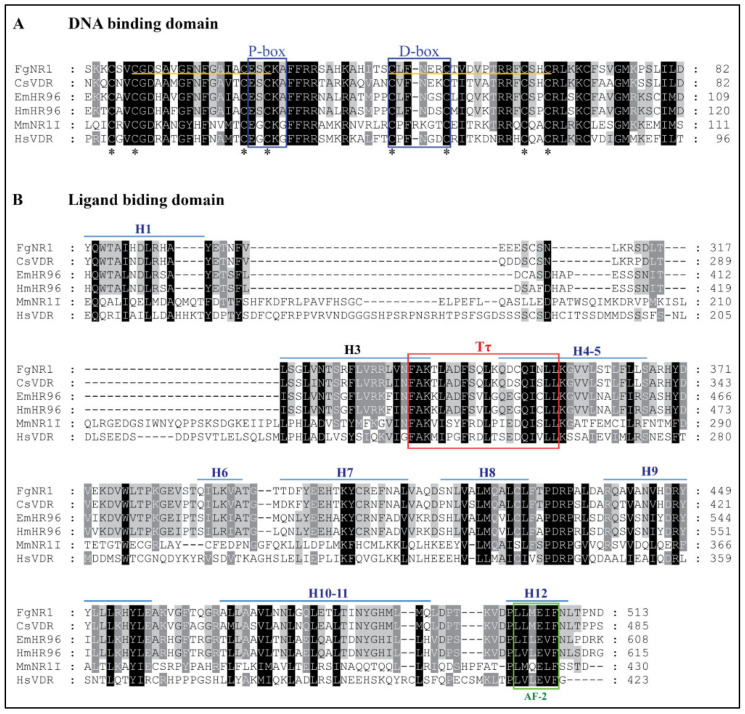
Multiple sequence alignment of FgNR1 sequence with homologs. The deduced amino acid sequence of nuclear receptor subfamily 1 homologs of F. ginantica (FgNR1), C. sinensis (CsVDR), E. multicularis (EmHR96), H. microstoma (HmHR96), M. musculus (MmNR1I), and H. sapiens (HsVDR) were aligned using MUSCLE. The identical and similar amino acids are shaded in black and gray, respectively. Gaps (−) are introduced to optimize homology. The number at the end of each line indicates residue position in the original sequence. (**A**) Sequence alignment of the DNA-binding domain (DBD). The two zinc-finger regions in DBD of FgNR1 are noted by the yellow underline. Asterisks indicate the conserved cysteines in zinc fingers. P-box and D-box are both indicated by a blue box. (**B**) Sequence alignment of the ligand-binding domain (LBD). The red box indicates the signature sequence (Tτ) of LBD. The putative autonomous activation domain (AF-2) is indicated by the green box. Helices (H1-12) are indicated by the blue overline. The GenBank accession numbers of the sequences are provided in Table A1.

Multiple amino acid sequence alignment of FgNR1 with homologs in the NR superfamily was analyzed to identify conserved motifs. The deduced amino acid sequence of nuclear receptor subfamily 1 homologs of F. ginantica (FgNR1), Clonorchis sinensis (CsVDR), Echinococcus multicularis (EmHR96), Hymenolepis microstoma (HmHR96), Mus musculus (MmNR1I), and Homo sapiens (HsVDR) were aligned using MUSCLE alignment. The result suggested that FgNR1 is highly conserved to the NR superfamily both in the DNA binding domain (DBD) (Figure 1A) and the ligand binding domain (LBD) (Figure 1B). Sequence alignment of the DNA-binding domain (DBD) revealed two conserved zinc-finger regions in DBD which indicated the conserved cysteines (P-box at position 22-26, and D-box at 40-47). However, the sequence alignment of the ligand-binding domain (LBD) indicated the signature sequence (Tτ) of NR-LBD at position 345-364. FgNR1 contains the putative autonomous activation domain (AF-2) and twelve helices (H1-12) as shown in Figure 1. The GenBank accession numbers of the sequences used in this study are provided in Table A1.

**Figure 2:**
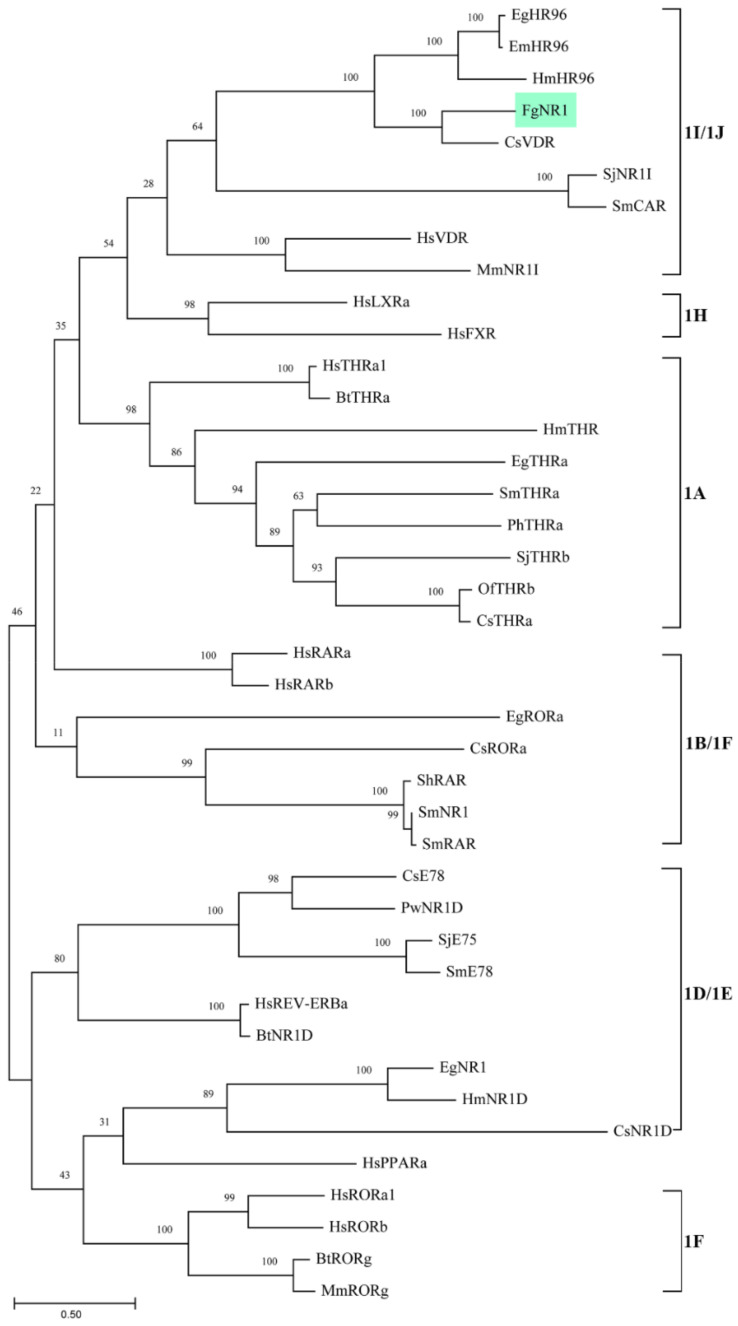
Phylogenetic analysis of the full-length of FgNR1 with nuclear receptor subfamily 1 homologs from other species. The tree was constructed with maximum likelihood method using MEGA11 program, 1000 bootstrap replicates. Numbers at the nodes represent the bootstrap values. The abbreviations used and GenBank accession numbers are provided in Table A1.

**Figure 3:**
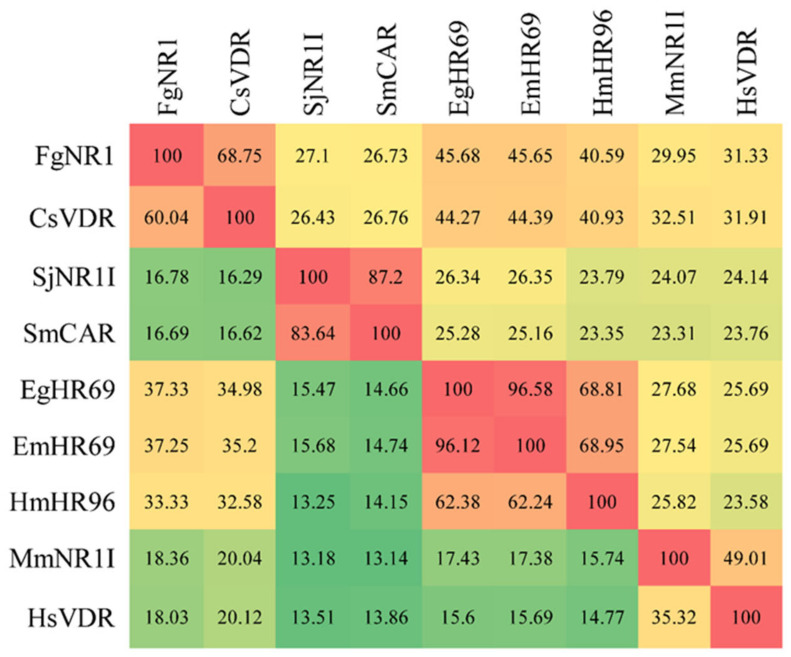
Comparative heatmap of FgNR1 with homologs. Percentage identity (lower left) and similarity (upper right) between FgNR1 and other members of NR1 subfamily were determined using the Ident and Sim program.

The phylogenetic tree was generated using 42 nuclear receptor orthologs from trematodes, cestodes, nematodes, and mammals belonging to family 1 subfamilies 1A to 1H. The tree revealed the evolutionary conservation of the FgNR1 to 1I/1J subfamily. The most closely related to FgNR1 was Clonorchis sinensis vitamin D receptor—CsVDR—that is located in the same clade but in a different separated branch. Moreover, FgNR1 has satisfactory conservation to other Platyhelminthic receptors, especially cestodes including Hymenolepis microstoma nuclear hormone receptor HR96; HmHR96, Echinococcus granulosus nuclear hormone receptor HR96; EgHR96, and Echinococcus multilocularis nuclear hormone receptor HR96; EmHR96 as shown in Figure 2. Even though the evolutionary phylogenetic tree suggested the conservation of FgNR1 to other NRs with high scores, the comparative heatmap demonstrated the deep details of differences by similarity and identity percentages of FgNR1 with other reported NR homologs as shown in Figure 3. 

### 3.2. Computational Modeling of FgNR1

**Figure 4:**
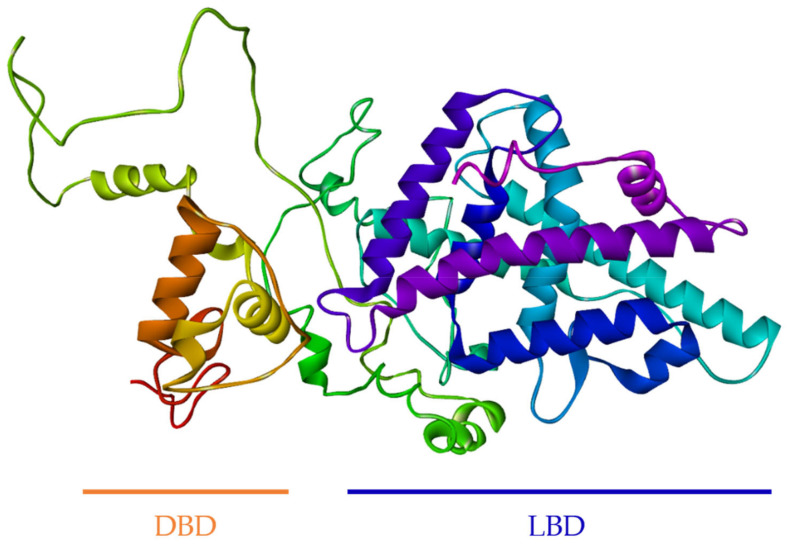
Tertiary structure model of FgNR1. The 3D structure of the full-length FgNR1 was constructed and refined by I-TASSER and GalaxyRefine server, respectively. The crystal structure of human Retinoic acid receptor RXR-alpha (PDB ID: 4NQA) was used as the template.

The secondary structure of full-length FgNR1 was predicted using the PDBsum server. It comprised 4 strands, 17 helices, 2 beta hairpins, 73 beta turns, and 20 gamma turns as illustrated in Figure S1. The 3D structure of the full-length FgNR1 was constructed and refined by I-TASSER and GalaxyRefine server. The crystal structure of human Retinoic acid receptor RXR-alpha (PDB ID: 4NQA) was used as the template. The conformation confirmed that the predicted structure of FgNR1 is closely related to human RXR-alpha, which is one of the major members of NR superfamily. The DNA binding domain (DBD) contains beta-hairpin coupled with two helices that specifically recognize target DNA (left side on Figure 4). Ligand-binding domain (LDB) was located on the other side of the model which comprised several helices and another hairpin for ligand-binding pocket formation. The Ramachandran plot (Figure S2) revealed that 82.2% of all residues were in the most favored regions, 13.5% in additional allowed regions, 2.6% in generously allowed regions, and only 1.7% in disallowed regions. This is a confirmation of the reliability of the 3D structural model of FgNR1. The 3D structure of FgNR1 is shown in Figure 4.

### 3.3. Molecular Cloning and Production of Recombinant FgNR1 (rFgNR1)

**Figure 5:**
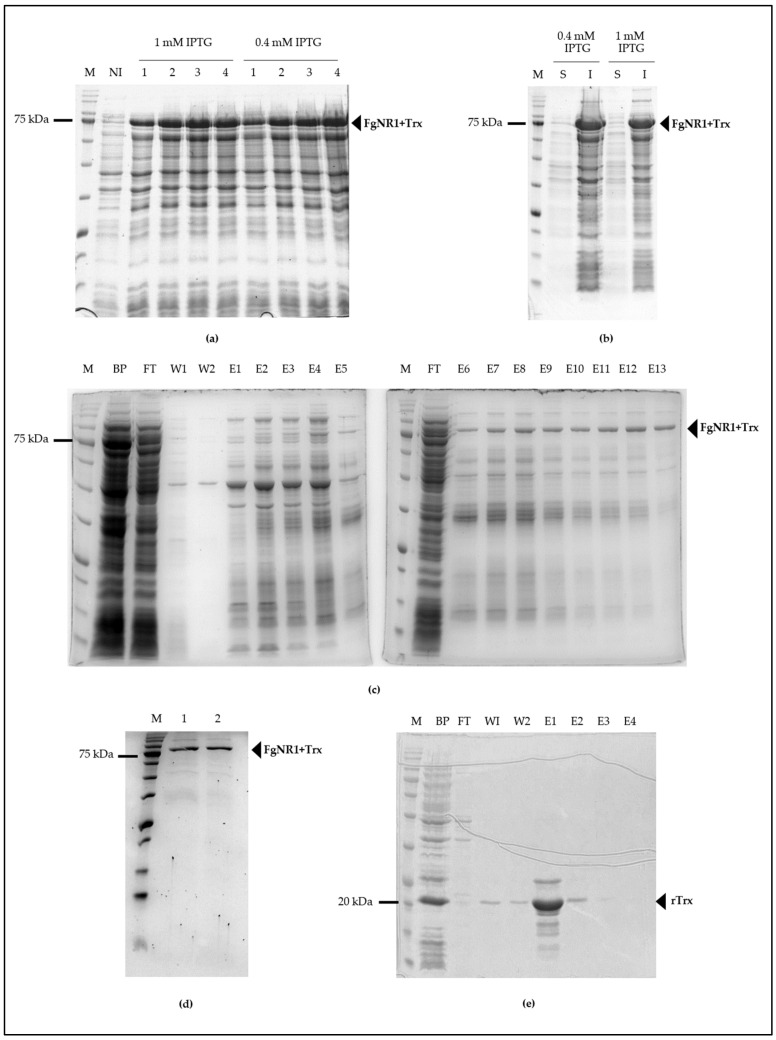
Production of recombinant proteins (rFgNR1+Trx and rTrx). (**a**) BL21(DE3) *E. coli* containing pET32a(+)/FgNR1 induced with 0.4 and 1 mM IPTG for 1 to 4 h. M: Tricolor Broad Range Prestained Protein Ladder (Vivantis, Malaysia); NI: non-induced; 1-4:1-4 h after induction. (**b**) Solubility of rFgNR1+Trx. S: soluble fraction; I: inclusion bodies. (**c**) Purification of rFgNR1+Trx under denaturing condition. BP: before purifying; FT: flow through; W1: wash fraction-1; W2: wash fraction-2; E1-E13: elution fraction 1-13 using various concentrations of imidazole from 10-250 mM. (**d**) Dialyzed and concentrated rFgNR1+Trx. Lane 1: dialyzed rFgNR1+Trx (20 μL), lane 2: concentrated rFgNR1+Trx (5 μL). (**e**) Purified rTrx under native condition using imidazole. BP: before purifying; FT: flow through; W1: wash fraction-1; W2: wash fraction-2; E1-E4: elution fraction 1-4.

The full-length FgNR1 PCR amplicon was successfully amplified from cDNA synthesized from total RNA of *F. gigantica* using FgNR1-specific primer as mentioned above. The PCR amplicon was then purified and ligated into pGEM-T easy vector and transformed into XL1 blue *E. coli* then processed to DNA sequencing. The transformants containing the corrected sequence were subcloned into pET32a(+) expression vector and then transformed into BL21(DE3) *E. coli* for recombinant protein expression. rFgNR1 was produced by inducing with 0.4 and 1 mM IPTG for 1 to 4 h at 37 °C in shaking incubator. rFgNR1+Trx was produced in an almost similar concentration after induction of various concentrations of IPTG. It was also produced in an equivalent concentration in 2 to 4 h after induction. rFgNR1 was expressed at molecular weight of 58.3 kDa plus 13.7 kDa of Trx fusion protein (rFgNR1+Trx: 72 kDa) as shown in Figure 5a. rFgNR1-Trx was expressed in the inclusion bodies as bioinformatics prediction (Figure 5b); as a result, FgNR1+Trx was purified by using Ni-Sepharose under a denaturing condition and finally eluted by using 8 M urea plus 250 mM imidazole in the last collected fraction as shown in Figure 5c. The dialyzed rFgNR1+Trx against PBS, pH 7.4, and concentrated rFgNR1+Trx, were illustrated in Figure 5d. rTrx was separately expressed from circular pET32a(+) and purified under native condition as shown in Figure 5e.

### 3.4. Detection of Native FgNR1 in Parasite Tissue and Extracts

**Figure 6:**
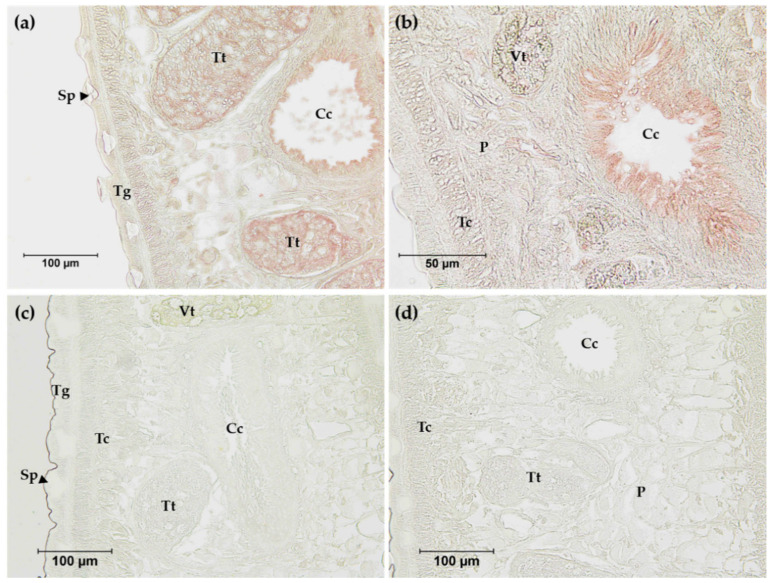
Immunolocalization of FgNR1 in adult *F. gigantica* tissue sections. (**a**) Anti-rFgNR1+Trx antibodies (pooled sera 1:200) at 200× magnification; (**b**) anti-rFgNR1+Trx antibodies (pooled sera 1:200) at 400× magnification; (**c**) preimmunized sera (pooled sera 1:200) at 200× magnification; (**d**) anti-rTrx antibodies (pooled sera 1:200) at 200× magnification. Cc: caecum; P: parenchyma; Sp: spine; Tc: tegumental cell; Tg: tegument; Tt: testes; Vt: vitelline gland.

The anti-rFgNR1+Trx was used to determine the distribution of native FgNR1 in the parasite tissue by immunolocalization. Anti-rTrx was used as a control of anti-fusion protein that should not detect any compartments in the parasite tissues as it was immunized together with rFgNR1. The results indicated that the positive signals (red) were concentratedly observed in the apical of caeca and testes of the parasite. FgNR1 was not detected in other organs including teguments, tegumental cells, parenchyma, or vitellaria. Pre-immunized sera were used as negative control. Moreover, a clear negative signal was observed with anti-rTrx in the parasite tissue. The immunohistochemistry results are illustrated in Figure 6.

**Figure 7:**
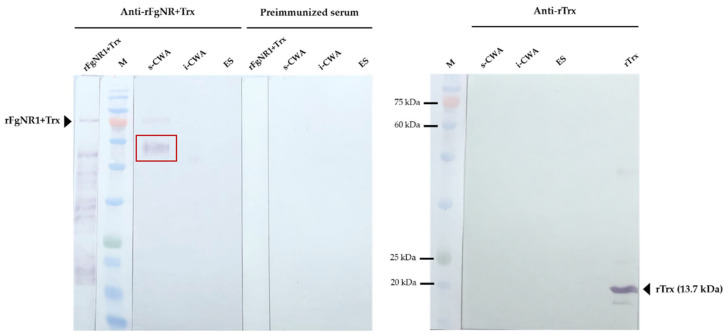
Western analysis of native FgNR1 in parasite extracts. All sera were diluted 1:200 in antibody diluent (1% BSA in TBS, pH 7.5). The red box indicated the expected band of native FgNR1, 58.3 kDa, in s-CWA protein extract of *F. gigantica*. Preimmunized sera and anti-rTrx clearly defined no unspecific detection in any parasite protein extracts.

Western analysis of native FgNR1 was carried out in parasite extracts including s-CWA, i-CWA, and ES products. A positive anti-rFgNR1+Trx signal was detected in the expected size of FgNR1 (approximately 60 kDa) for CWA-s, but not for i-CWA nor ES as shown in Figure 7. It also demonstrated the specific signal in the rFgNR1+Trx that is used to immunization. This result corresponded to the immunolocalization of FgNR, that the positive signals on parasite tissues appeared from only anti-rFgNR1 and excluded anti-rTrx.

### 3.5. Bile Stimulated FgNR1 mRNA Expression

**Figure 8:**
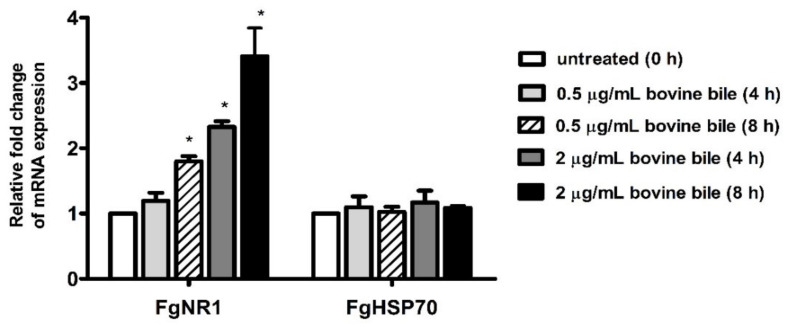
Semiquantitative real-time PCR of FgNR1 and FgHSP70. The graph represents the significant differences of FgNR1 mRNA expression 4 h after incubation with 2 μg/mL, and 8 h after incubation with 0.5 and 2 μg/mL of bovine bile normalized with FgTUB. FgHSP70 showed no significant difference among the groups. (* represented significant difference at *p*-value < 0.001).

Semiquantitative real-time PCR of FgNR1 derived from cultured parasites with bovine bile suggested that FgNR1 mRNA expression could be stimulated by bovine bile in a dose-dependent manner. The mRNA expression of FgNR1 at 4 h after incubation demonstrated that a low concentration of bile (0.5 μg/mL) could not significantly alter FgNR1 mRNA expression when compared with untreated worms at 0 h (relative fold change of 1.193 ± 0.125; mean ± SD), while a high concentration of bile (2 μg/mL) significantly upregulated FgNR1 mRNA expression (*p* < 0.001) with the relative fold change of 2.327 ± 0.087. Additionally, FgNR1 mRNA expression at 8 h after incubation with both low and high concentrations of bile demonstrated the significant upregulation in different levels with relative fold changes of 1.800 ± 0.080 (*p* < 0.001) and 3.407 ± 0.434 (*p* < 0.001), respectively. In contrast, the FgHSP70, irrelevant protein, was not significantly different in all groups. The result is shown in Figure 8.

## 4. Discussion

Nuclear receptors (NRs) are a huge family of nucleoproteins accumulating in the nucleus of the metazoans regulating many cell activities, such as cell proliferation, differentiation, reproduction, and metabolisms [13,14]. NRs are recognized as the ligand-regulated transcription factors that are mainly activated by steroid hormones, such as estrogen and progesterone, also various other lipid-soluble and steroid-like molecules, including retinoic acid and thyroid hormone [53]. Unlike most intercellular messengers, the ligands directly interact with nuclear receptors inside the nucleus by crossing the plasma membrane without having cell surface receptors [35]. The structure of NRs consists of two major conserved domains; DNA binding domain (DBD) and ligand-binding domain (LBD). DBD is a highly conserved domain among NRs that consists of zinc-finger proteins forming two helices, P-box and D-box, whereas LBD is more variable [15,54,55]. In the parasitic organisms, the first identified NR was reported from *Schistosoma mansoni* [56] and later in *Taenia crassiceps*, *Opistorchis felineus*, and *Echinococcus* spp. [26,27,35,56,57,58]. The available transcriptome of *F. gigantica* allowed us to find NRs in the adult parasite including FgNR1 [59]. The FgNR1 used in this present study was carried out based on the sequence annotated existing in the Genbank (accession number: TPP57711.1). The bioinformatics analysis demonstrated that the FgNR1 sequence reported in the database comprises a correct coding sequence containing 523 amino acid residues without unusual residues. The FgNR1-DBD consisted of two zinc-finger regions called P-box and D-box that are conserved among the NR subfamily [13]. P-box domains distinguish NRs and discriminate the central recognition element of DNA [60]. In general, the P-box contains EGCKG-sequence (EGG) [61], but interestingly, FgNR1 has a different P-box sequence that contains ESCKA (ESA), which is similar to CsVDR, EmHR96, and HmHR96. We hypothesized that FgNR1 will trigger similar DNA targets as CsVDR, but this is still unknown. Another DNA recognition motif is called D-box, which consists of a 5-amino acid loop that defines a strong dimerization interface for homodimer formation and contributes to heterodimer stabilization [61,62]. The D-box of FgNR1 contains LFNER sequence, which differs from other members of this subfamily. This finding suggested that FgNR1 could interact with different targets from the others, but this need to be identified further. Another domain is the ligand-binding domain, LBD, in which a higher variable region than DBD depends on the ligand that binds to each NR. However, LBD should consist of the signature sequence (Tτ) that recognizes the hexanucleotide response elements, which are critically used for forming of the ligand-binding pocket [54,55,63]. The result demonstrated that FgNR1 contains the Tτ signature sequence which confirmed that FgNR1 could be stimulated by the nuclear receptor-specific ligands.

Additionally, the tertiary structure of FgNR1 that was generated by using the crystal structure of human retinoic acid receptor RXR-alpha as the template according to the complete crystal structure has been clearly reported [64]. The conformation confirmed that FgNR1 has the predicted structure closely related to human RXR-alpha, which is one of the major members of the NR superfamily. FgNR1-DBD contains beta-hairpin coupled with two helices that specifically recognized target DNA. The ligand-binding domain (LDB) was located on the other side of the model, which comprised several helices and another hairpin for ligand-binding pocket formation. Our findings demonstrated that FgNR1 structural prediction could be used for facilitating drug design using in silico docking in the future.

As mentioned earlier, the NRs superfamily consists of six major families. The phylogenetic analysis demonstrated that FgNR1 was classified into family 1, in which closely related to subfamily 1I/1J. Moreover, FgNR1 has a highly evolutionary relationship to the NRs of flatworms, both trematodes and cestodes including *C. sinensis*, *Echinococcus* spp. and *Schistosoma* spp. In humans, are used as a template for NRs classification, subfamily 1I/1J consists of vitamin D receptor (VDR), pregnane X receptor, and constitutive androstane receptor, which could be activated by both exogenous and endogenous ligands [15,65,66]. The closest evolution related to FgNR1 was CsVDR which is located in the same clade but in a different separated branch, followed by HmHR96, EgHR96, and EmHR96. CsVDR was the only computational predicted gene existing in the transcriptome of *C. sinensis* without any functional characterization [67]. In this clade, only EmHR96 has been characterized, which has shown an important role in hormonal host–parasite cross-communication mechanisms during an infection; unfortunately, the deep details of its function as well as its molecular interaction remain under investigation [68]. Interestingly, another subfamily next to 1I/1J with a lower score was 1H, which comprises fernesoid X receptor (FXR) which is stimulated by bile salts [69]. Even though FgNR1 stated high evolutionary conservation to the NRs in 1I/1J subfamily, the heatmap analysis suggested that FgNR1 existed with an intermediate identity and similarity percentages with the highest scores to CsVDR by 60.04 and 68.75%, respectively. These computational predicted results suggested that FgNR1 contains different sequences that could be stimulated by either a ligand that interact with subfamily 1I/1J, such as vitamin D3, endobiotics, and xenobiotics, or different ligands that activate other NRs, such as oxysterols, sterols, fatty acids, also bile salts.

Full-length FgNR1 was successfully expressed as an insoluble protein coupled with rTrx fusion protein in a prokaryotic expression system at the molecular weight of 58.3 kDa plus 13.7 kDa of rTrx, as predicted. Unfortunately, the fusion protein, rTrx, could not be separately cleaved by enterokinase even at the highest concentration and after 72 h of incubation. So, the recombinant proteins should be used together for immunization which is called rFgNR1+Trx. rFgNR1-Trx was successfully purified under denaturing condition and used as an immunogen. rTrx from empty vector, pET32a(+), was expressed separately and purified under native condition as it was produced soluble. The polyclonal antibodies from mice against rFgNR1+Trx and rTrx were verified by indirect ELISA using rFgNR1+Trx and rTrx before use in the native protein detection. The immunolocalization of native FgNR1 illustrated that the positive signals were observed mainly in the apical of caeca and testes of the parasite. The anti-rTrx was used together with anti-rFgNR1+Trx, but interestingly, no signal could be detected. This result strongly suggested that the positive signal was specifically defined by only anti-rFgNR1. The locations that found the positive signals of native FgNR1 in our study were different from *S. mansoni*, which is expressed in the esophageal gland [70], and from *E. granulosus*, which is evenly distributed throughout all tissues of both the adult worms and PSCs [26]. This could be possible due to the different biological life cycle of each parasite that inhabits different environments. Interestingly, our findings suggested that FgNR1 might be involved in the reproduction of the parasite, especially in the male reproductive system; however, this hypothesis must be proved in the future. Moreover, the native FgNR1 that is concentratedly expressed in the caeca suggests that this would be another target organ of bile salt that comes with dietary nutrients [1,4,71]. Western analysis of native FgNR1 in parasite extracts confirmed the immunolocalization result of FgNR1. The soluble CWA (s-CWA) fraction contains proteins inside the cell, both in the cytoplasm and the nucleus, except for proteins that are intact to inclusion bodies, such as transmembrane and membrane-bound proteins [72,73,74]. The Western blot result demonstrated that native FgNR1 was expressed in the correctly predicted size, 58.3 kDa. This part of the results strongly suggested that FgNR1 will be the important molecule in parasite metabolism, especially in digestion and reproduction.

From all above results, we would like to confirm our hypothesis that FgNR1 could be activated by bile, which contains bile salts. Interestingly, the semiquantitative real-time PCR demonstrated that the worms that culture in the culture medium containing bile indicated a higher level of FgNR1 mRNA expression when compared with the culture medium without bile in a time-dependent manner. This result suggested that bile components, especially bile salts, will be the key molecule that stimulate FgNR1, not only on the transcriptional level but also in terms protein expression, as this has occurred in other bile salt nuclear receptors [75,76]. The interaction of bile salts, especially lithocholic acid (LCA), with human vitamin D receptor (VDR) triggers that hypothesis due to FgNR1 being evolutionarily conserved to VDR [77]. Moreover, the finding that FgNR1 will be the molecule that modulates the reproductive system of *F. gigantica* corresponds with the study on *S. mansoni*, in which bile salts regulated oviposition with unknown mechanisms [22]. However, the interaction of bile salts with FgNR1 must be investigated on a deeper level, especially in terms of molecular mechanisms. The understanding of this mechanism will be useful for the development of FgNR1 to be an effective drug target in the future.

## 5. Conclusions

In conclusion, FgNR1 was the first nuclear receptor from trematode parasites that potentially interacts with bile components—and likely bile salts. It has been molecularly characterized, cloned, and produced as recombinant protein for polyclonal antibody production. The immunolocalization and Western analysis suggested that FgNR1 may play the major biochemical role in the reproductive and digestive systems of the parasite. The molecular mechanisms of FgNR1 needs to be further investigated due to it having high possibility to be an effective drug target for fasciolosis and other associated diseases.

## Data Availability

Not applicable.

## Supplementary Materials

The following supporting information can be downloaded at: https://www.mdpi.com/article/10.3390/pathogens11121458/s1, Figure S1: Secondary structure of FgNR1; Figure S2: Ramachandran plot of FgNR1.

## Appendix A

**Table A1:** Abbreviations and GenBank/accession numbers of NR homologs were used in this study.

Abbreviation	Species *	Protein Name	GenBank/Accession No.
FgNR1	*F. gigantiga*	Nuclear receptor subfamily 1	TPP57711.1
HsTHRa1	*H. sapiens*	Thyroid hormone receptor-α1	CAA38749.1
HsRARa	*H. sapiens*	Retinoic acid receptor-α	CAA29829.1
HsRARb	*H. sapiens*	Retinoic acid receptor-β	CAA30262.1
HsPPARa	*H. sapiens*	Peroxisome proliferator-activated receptor-α	AAA36469.1
HsREV-ERBa	*H. sapiens*	Reverse-Erb-α	AAA52335.1
HsRORa1	*H. sapiens*	Retinoic acid-related orphan-α1	AAA62658.1
HsRORb	*H. sapiens*	Retinoic acid-related orphan-β	CAA69929.1
HsLXRa	*H. sapiens*	Liver X receptor-α	AAA85856.1
HsFXR	*H. sapiens*	Farnesoid X receptor	AAB08107.1
HsVDR	*H. sapiens*	Vitamin D receptor	AAA61273.1
BtNR1D	*B. taurus*	Nuclear receptor subfamily 1D	NP_001071568.1
BtRORg	*B. taurus*	Retinoic acid-related orphan-γ	NP_001076920.1
BtTHRa	*B. taurus*	Thyroid hormone receptor-α	NP_001039794.1
MmNR1I	*M. musculus*	Nuclear receptor subfamily 1I	NP_035066.1
MmRORg	*M. musculus*	Retinoic acid-related orphan-γ	NP_035411.2
OfTHRb	*O. felineus*	Thyroid hormone receptor-β	AFM38060.1
CsTHRa	*C. sinensis*	Thyroid hormone receptor-α	KAG5441315.1
CsE78	*C. sinensis*	Ecdysone-induced protein 78C	KAG5445165.1
CsNR1D	*C. sinensis*	Nuclear receptor subfamily 1D	GAA57457.1
CsRORa	*C. sinensis*	Retinoic acid-related orphan-α	GAA58146.1
CsVDR	*C. sinensis*	Vitamin D receptor	KAG5451015.1
ShRAR	*S. haematobium*	Retinoic acid receptor	XP_035588303.1
SjNR1I	*S. japonicum*	Nuclear receptor subfamily 1I	TNN17786.1
SjE75	*S. japonicum*	Nuclear hormone receptor E75	KAH8876702.1
SjTHRb	*S. japonicum*	Thyroid hormone receptor-β	AFP95236.1
SmNR1	*S. mansoni*	Nuclear receptor subfamily 1	AAR29357.1
SmTHRa	*S. mansoni*	Thyroid hormone receptor-α	AAR29358.1
SmE78	*S. mansoni*	Ecdysone-induced protein 78	AAR30507.2
SmRAR	*S. mansoni*	Retinoic acid receptor	XP_018649430.1
SmCAR	*S. mansoni*	Constitutive androstane receptor	AAV80235.1
PwNR1D	*P. westermani*	Nuclear receptor subfamily 1D	KAA3676691.1
PhTHRa	*P. heterotremus*	Thyroid hormone receptor-α	KAF5398468.1
EgRORa	*E. granulosus*	Retinoic acid-related orphan-α	KAH9284012.1
EgHR96	*E. granulosus*	Nuclear hormone receptor HR96	CDS22803.1
EgTHRa	*E. granulosus*	Thyroid hormone receptor-α	CDS24335.1
EgNR1	*E. granulosus*	Nuclear receptor subfamily 1	ART84255.1
EmHR96	*E. multilocularis*	Nuclear hormone receptor HR96	CDS36018.1
HmNR1D	*H. microstoma*	Nuclear receptor subfamily 1D	CDS29812.1
HmHR96	*H. microstoma*	Nuclear hormone receptor HR96	CDS28331.1
HmTHR	*H. microstoma*	Thyroid hormone receptor	CDS30783.1

* Abbreviations: *F. gigantica*: *Fasciola gigantica*; *H. sapiens*: *Homo sapiens*; *B. taurus*: *Bos taurus*; *M. musculus*: *Mus musculus*; *O. felineus*: *Opisthorchis felineus*; *C. sinensis*: *Clonorchis sinensis*; *S. haematobium*: *Schistosoma haematobium*; *S. japonicum*: *Schistosoma japonicum*; *S. mansoni*: *Schistosoma mansoni*; *P. westermani*: *Paragonimus westermani*; *P. heterotremus*: *Paragonimus heterotremus*; *E. granulosus*: *Echinococcus granulosus*; *E. multilocularis*: *Echinococcus multilocularis*; *H. microstoma*: *Hymenolepis microstoma*.
