# Supplementary material for: Molecular Cloning and Characterization of a Fasciola gigantica Nuclear Receptor Subfamily 1 (FgNR1)
Source: Pathogens. 2022 Dec 1;11(12):1458. doi: 10.3390/pathogens11121458 (PMC9787296; doi:10.3390/pathogens11121458)
Supplement: Supplementary file 1 [file pathogens-11-01458-s001.zip › pathogens-1974723-supplementary.pdf]

pathogens MDPI

Supplementary Materials

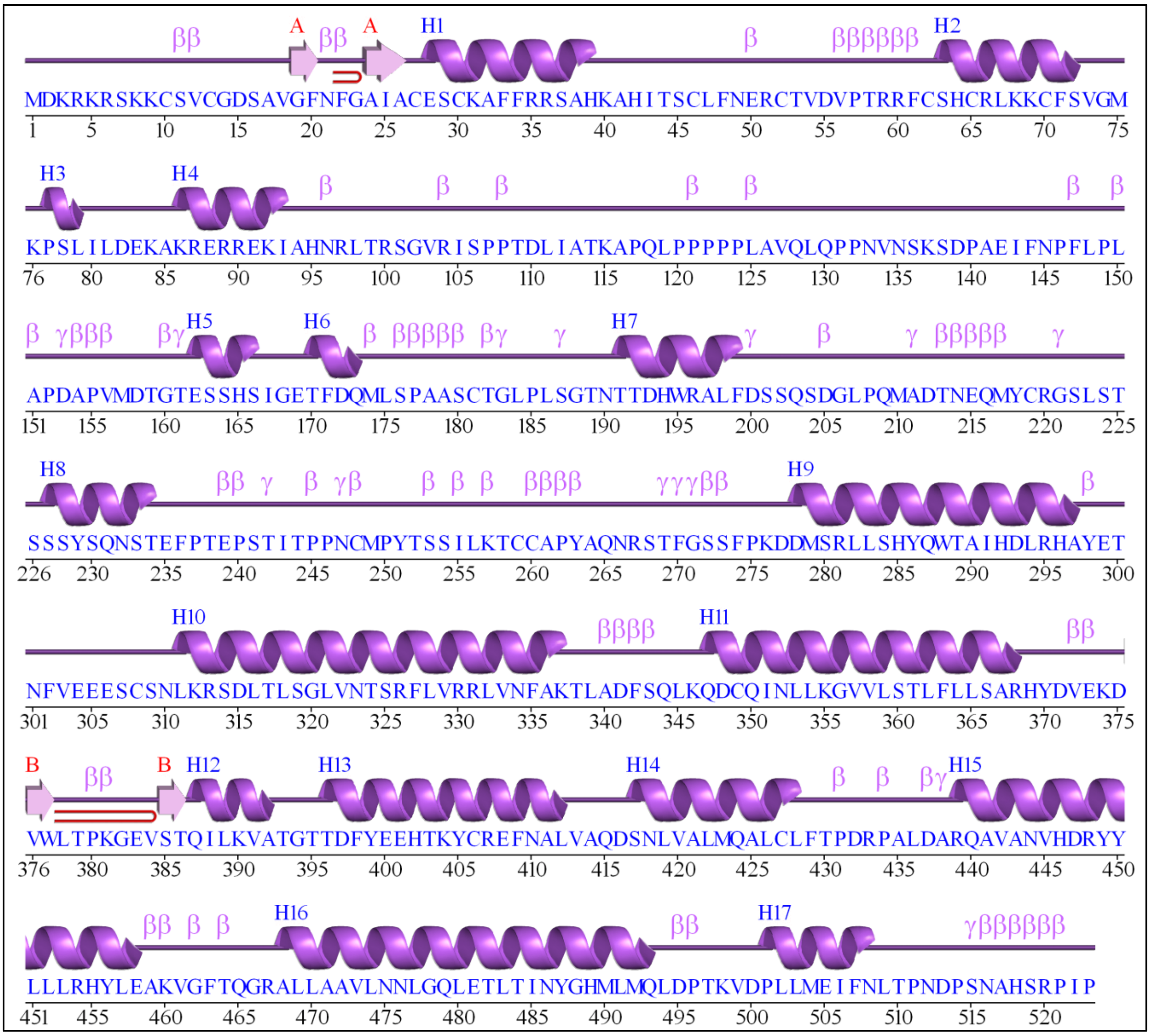

Figure S1. Secondary structure of FgNR1 predicted by PDBsum server showing 4 strands, 17 helices, 2 beta hairpins, 73 beta turns, and 20 gamma turns.

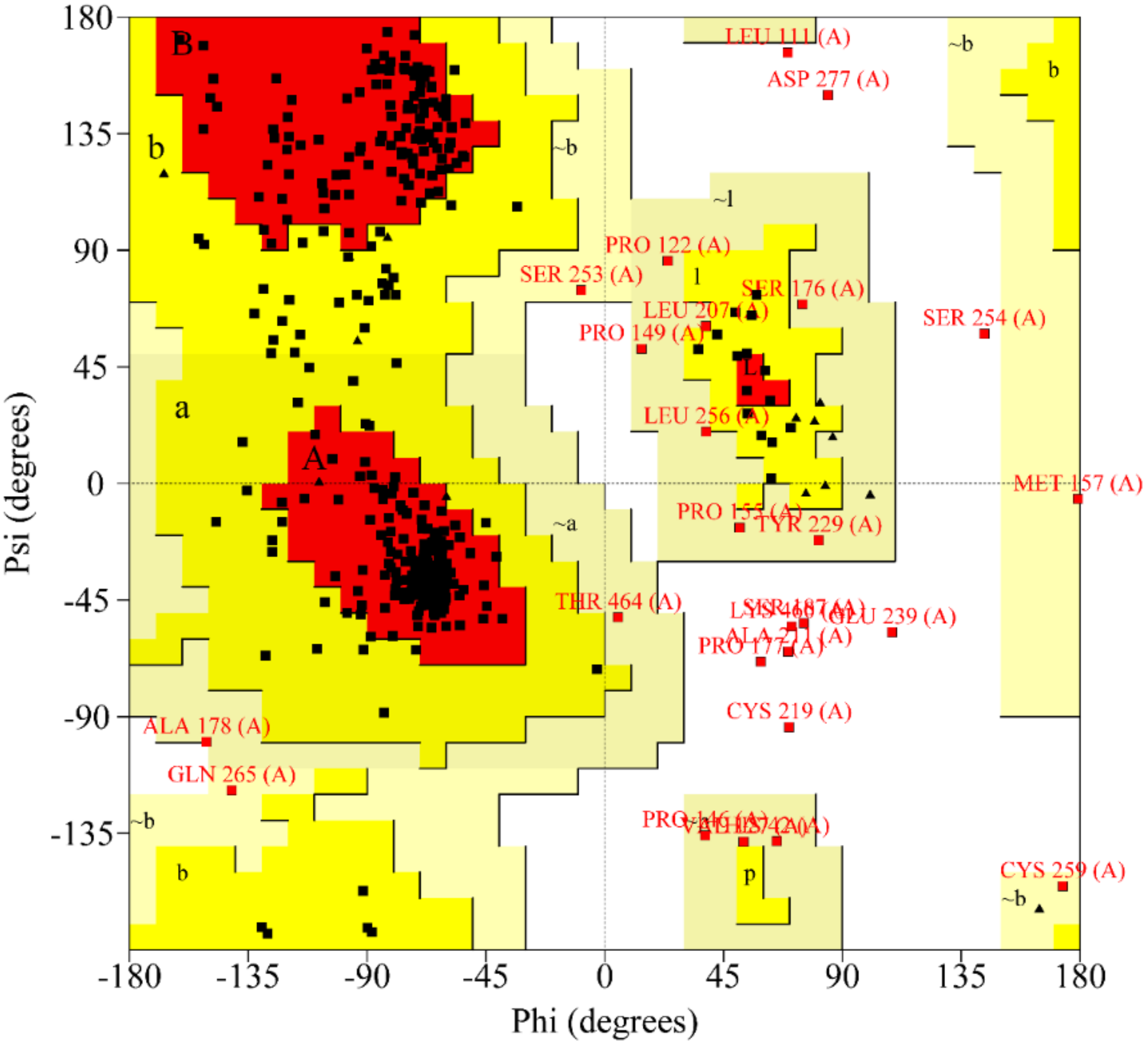

Figure S2. Ramachandran plot of FgNR1, produced by PROCHECK (validation of 3D structure). Ramachandran plot revealed that 82.2% of all residues were in most favored regions, 13.5% in additional allowed regions, 2.6% in generously allowed regions, and only 1.7% in disallowed regions.
